# Key Drivers for Non-Centrifugal Sugar Cane Research, Technological Development, and Market Linkage: A Technological Roadmap Approach for Colombia

**DOI:** 10.1007/s12355-022-01200-9

**Published:** 2022-09-01

**Authors:** Diego Hernando Flórez-Martínez, Carlos Alberto Contreras-Pedraza, Sebastian Escobar-Parra, Jader Rodríguez-Cortina

**Affiliations:** Corporación Colombiana de Investigación Agropecuaria–AGROSAVIA, Km 14 Vía Mosquera–Bogotá, Mosquera, 250047 Cundinamarca Colombia

**Keywords:** Non-centrifugal sugar cane, Technological surveillance, Competitive intelligence, Patent analysis, Functional foods, Bioactive compounds, Technological roadmap

## Abstract

Food science innovation depends on consumers’ needs and is currently seeking functional food with health effects. Non-centrifugal cane sugar (NCS) is known for its potential health effects, but there is a lack of holistic analysis on technological advancement and socio-economic and market trends for decision-making in the development of the technology. The aim of this article was to analyse the research trends, recent patents, and market trends and niches for NCS to structure an NCS technological roadmap. Scientometric, bibliometric methods, and global and local market information on NCS were used. Comprehensive analysis of the worldwide research trends and patents on NCS processing and of the growth of the main niche markets for Colombian NCS exports in the last five years was conducted. Finally, with the information obtained, an NCS technological roadmap was structured, which can be used as a tool for planning innovation processes and supporting the development of new research using market information and new norms forged by the COVID-19 pandemic for Colombian case. Furthermore, the methodological design could be used for other NCS producer countries.

**Supplementary Information:**

The online version contains supplementary material available at 10.1007/s12355-022-01200-9.

## Introduction

Functional food has been at the centre of research and drives the growth of the market. This is due to recent technological advancement and socio-economic trends that increase the consumer demand for healthy natural foods with powerful health benefits. The health benefits of functional foods are derived from bioactive compounds (phytochemicals, vitamins, and peptides) found naturally or formed during processing (Butnariu and Sarac 2019). Furthermore, researchers have conducted different studies seeking to find solutions for consumers' demand and interest in health-promoting foods (Tadesse and Emire 2020).

Currently, the perception of food has shifted from providing essential nutrients for sustaining life to preventing or curing various diseases. This trend accompanies population lifestyle changes and socio-economic situations (Betoret et al. 2011). These are the key determining and driving forces for the technological development and production of functional foods in the global market.

The size of the functional food market was valued at $177,770.00 million in 2019 and is expected to reach $267,924.40 million by 2027, registering a compound annual growth rate (CAGR) of 6.7% from 2021 to 2027. Despite the trade restrictions during COVID-19, the pandemic has positively influenced the growth of the functional food market. Functional foods have broad potential for preventing the mechanisms of viral infection and modulating immune responses (Alexander Haslberger et al. 2020). Therefore, people are highly conscious and wish to improve their health and immune power by consuming highly nutritious food. Furthermore, the COVID-19 pandemic has accelerated current market growth trends in the food and nutraceutical sector. This trend has forced businesses to address the challenges and opportunities created by COVID-19, thereby accelerating innovation across the food industry (Galanakis et al. 2021).

Sugar cane juice is principally used for sugar crystal production as a sweetener. However, there has been considerable negative attention on sugars because of their relationship with obesity (Eggleston et al. 2021). This has led to substantial growth in the use of “unrefined” and “natural” products made from sugar cane, as in the case of non-centrifugal cane sugar (NCS). Jaffé (2015) reported the different nutritional and bioactive components (phenolic and flavonoid compounds) of sugar cane juice that are good for human health (Velasquez et al. 2019).

Flórez-Martínez et al. (2021) constructed a “big picture” for future project focalization in NCS research. This study confirmed interest in NCS as a source of bioactive compounds that could lead to the promotion of two principal lines of research focused on (*i*) functional and nutraceutical food and *(ii*) NCS as a healthy compound in pharmaceutical and cosmetic products.

The NCS market has been growing since the demand for functional food and healthy ingredients in the food, cosmetic and pharmaceutical industries became a trending topic in food science. Nutraceutical alternatives to refined sugar in juices, jams, bakery products, and canned food comprise a competitive market for natural (stevia) and artificial (mannitol) inputs. Furthermore, NCS can be a key alternative due to the availability of its vitamin, mineral, flavonoid, polyphenol, and other bioactive compounds.

In the same way, the development of a new processing form to preserve functional compounds is necessary. Therefore, information and knowledge management for scientific and academic research is an important tool (Flórez-Martínez et al. 2021) that allows us to understand the food science trends, such as the trends in the development of the technology.

Scientometrics and bibliometric analyses have been used recently to support scientific and technological surveillance activities, trend analysis, and science evolution through time slips (Garcia-Garcia and Rodríguez 2018). These analyses are not limited to scientific article data; instead, patent data are used for technological development in both product and service evaluations (Rotolo et al. 2017). Furthermore, patent analysis in food and agricultural research contributes to drawing research routes ranging from basic passes to applied and adaptive research. Some studies, such as Orozco Colonia et al. (2020) and Silveira et al. (2018), used literature reviews and sequential technological prospects using patent mapping to conduct research on food trends in omega-3 microbial oils and bioactive molecule content, respectively.

Similarly, the demand for functional foods in the global market has increased the interest in mapping and tracing the advancement of processing and production technologies and people's awareness of the importance of bioactive compounds as health-promoting ingredients. Therefore, the aim of this work was to present comprehensive analysis based on technology development to produce the current Colombian NCS market.

## Materials and Methods

The proposed methodological framework (Suppletory Material, Fig. 1) comprises five phases related to information search, data recovery, data analysis, and technological roadmap design based on Choo (2001); Flórez-Martínez et al. (2021); Phaal et al. 2004); and, Porter (2005).

### Search Strategy–Phase 1

This phase embraces the principles for structural equation design and data recovery from Scopus® (scientific publications), PatentInspiration® (patents), Legiscomex (trade information for Colombian case), and EMIS® (Market reports for functional foods). The data recovered from Scopus include records from January 2020 to July 2021 that represent recent NCS advances and records from 1997 to 2021 associated with functional food research trends. Furthermore, the data recovered from PatentInspiration® include records from 2000 to 2021. Global market trend data and Colombian NCS foreign trade data from 2016 to 2021 were recovered using specific keywords related to functional foods and NCS tariff headings on EMIS® and Legiscomex, respectively (Suppletory Material, Table 1).

### Research Lines on NCS, Recent Advances–Phase 2

Based on data recovered from phase 1, three specific analyses based on scientometric analysis techniques were developed. Thematic map and bibliographic coupling map analysis using Bibliometrix® (Aria and Cuccurullo 2017; Rui et al. 2018) contribute to the categorization of the research topics and key documents. Finally, cooccurrence network analysis was conducted using VOSviewer® according to Kostoff and Schaller 2001, and Rodríguez-Salvador et al. (2017) to identify the relationships among thematic clusters and key topics. Moreover, based on the research lines identified by Flórez-Martínez et al. (2021), publications were categorized on each research line based on title and abstract analysis: Line 1–Uses of NCS as a functional and nutraceutical food and food input. Line 2–NCS as a healthy compound in pharmaceutical and cosmetic products. Line 3–NCS production process optimization and greening. Line 4–NCS production by-products.

### Patent Analysis–Phase 3

Patent analysis contributes to the technological development mapping of new products, processes, devices, and enhancements of previous technology related to economic and productive sectors. The patent data recovered in phase 1 include information on the contributions of scientific knowledge to product and process innovation related to NCS research niches. Using the PatentInspiration® interface, two analyses were conducted: (i) Modifier analysis, which comprises the key attributes of technologies that increase, decrease, or change a current challenging situation; and (ii) A value equation, which presents the attributes of the patents related to performance, negative and positive factors, uses, and cost influences. Furthermore, using PatentInspiration metadata, two different cooccurrence networks were designed using title–abstract text and international patent classification codes (IPCs), respectively, on VOSviewer®. Additionally, patents were classified on each research line based on title and abstract analysis.

### Global and Local Market Niches–Phase 4

Colombian trade potential was analysed using detailed Legiscomex® data identifying the destination countries, clients (importing companies), exported values, and exported quantities. Furthermore, clients were classified based on specific market niches, due to commercial activity (declared on websites). Moreover, the EMIS database was consulted for market trend reports on smart foods and nutraceutical beverages. Finally, each client was related to a specific research line.

### Holistic Analysis Technological Roadmap–Phase 5

This phase comprises the results from phases 2, 3, and 4 using roadmapping methodology designed by Daim et al. (2018) and Phaal et al. (2004). The holistic analysis summarizes the relations between scientific research, technological development, and market niches in short-, medium-, and long-range planning. The roadmap comprises four layers: (i) Layer 1, scientific advances related to research papers from Scopus for each research line from 2020 to 2021; (ii) Layer 2, technologies identified from PatentInspiration registries; (iii) Layer 3, potential products based on NCS uses; and (iv) Layer 4, market niches. The layers and the relations of the items on each layer, including both intra- and interconnections, are time dependent.

## Results

In this section, the results are presented sequentially, according to the methodological design from Sect. 2.

### Search Strategy–Phase 1, Results

The search equations used in this work comprise the criteria presented on Sect. 2.1 (Suppletory Material, Table 2). The results for each search equation were: (1) Scopus®, 281 scientific publications registries from 2017 to 2021. PatentInspiration®, 608 related patents; (2) Scopus® 573 scientific papers focused on functional foods trends; (3) Scopus® 19 scientific paper focused on NCS as a functional food; (4) Legiscomex®, 4,285 international trade records for Specific tariff heading, 1,701,130,000 cane sugar mentioned in subheading note 2 of Chapter 17, from 2017 to 2021; and (5) Emis® twelve sectorial reports on nutraceutical food and functional beverages.

### Research Trends and Recent Advances–Phase 2, Results

#### Research Trends on Functional Foods

Technological trend analysis contributes to new research lines, product and service lines, and niche markets for researchers, industries, and markets, respectively. This premise has been previously aborded by Bigliardi and Galati (2013) who focused on functional foods such as fortified, enriched, altered, and enhanced foods as a long-term trend that integrates health, nutrition, and technology. Farias et al. (2019) proposed a review of prebiotic use in foods ranging from synthesis to application until health benefits are derived from consumption. Fang and Bhandari (2010) reviewed encapsulation technologies for polyphenols, bioactive molecules present in foods. Trend studies on functional foods on Scopus® (Eq. 4) analysed on VOSviewer encompass the scientific landscape for current and future research. Furthermore, a specific analysis using Eq. (3) allowed the identification on NCS research as a functional food. Figure 2 (Suppletory Material) presents the global landscape of functional foods as a referential framework for future works on NCS vs. the current studies on NCS as a functional food.

The functional food scientific landscape merges eight thematic clusters: (i) The red cluster, including the functional ingredients used in food products such as beverages, processed foods, emulsions, and proteins; increasing physicochemical, antioxidant, and rheological properties; enhanced nutritional qualities; and processing factors. NCS as a functional ingredient has been used as a sugar substitute on beverage industries due to its fructose, ascorbic acid, and minerals content (Cervera-Chiner et al. 2021); (ii) The green cluster, including dietary supplements based on functional foods contributing to metabolic function such as vitamin D, tocopherol, carotenoids, zinc, iron, calcium, magnesium, potassium, and bioactive compounds for cancer prevention, cholesterol control, glucose blood level control, intestinal flora control, etc. NCS as a dietary supplement ingredient confers antioxidative properties (Zidan and Azlan 2022); (iii) The blue cluster, including antioxidant function from bioactive compounds such as polyphenols, anthocyanins, amino acids, peptides, and flavonoids in both primary food products and by-products. NCS phenolic compounds promote antioxidant activities (Seguí et al. 2015); (iv) The yellow cluster, including food industries using functional foods to promote nutritional value, address consumer preferences, promote health, supplement vitamins and minerals and align nutrition policies; NCS is considered a key component for food reformulation and food safety in some countries (De Maria 2013; Revathi and Rani 2021); (v) The violet cluster, including plant extracts used as nutraceutical additives and phenol derivatives for anti-inflammatory, antineoplastic, antiproliferative, antifungal, and antimicrobial activities. NCS has been used together with plant extracts for hot beverages infusions (Singh et al. 2008); (vi) The cyan cluster, including probiotic and prebiotic compounds that enhance food matrix development, food quality, nutritional characteristics, and food production as a symbiotic agent. NCS flavonoids content enhances beverages and bakery products formulation (Elegado et al. 2016; Ali et al. 2019); (vii) The orange cluster, including major functional food topics related to drug additive discovery, handling, the supply chain, food science and technology issues, health impacts, and market and research trends. Some studies promote NCS as an active ingredient with anticancerogenic, antidiabetic, and anti-Alzheimer disease properties (Cifuentes et al. 2021; Revathi and Rani 2021); and (viii) The cluster, including food applications in the cosmetic, pharmaceutical, and food industries. Brown sugar has been widely used for cosmetic industries (Chinnadurai 2017; Bahari et al. 2021).

#### NCS Research Trends

NCS research trends according to Ebadi and Azlan (2021); Flórez-Martínez et al. (2021); Jaffé (2015); Velasquez et al. (2019); Zidan and Azlan (2022) comprise four key research lines (Suppletory Material, Fig. 3):

Research Line 1–NCS uses as a functional and nutraceutical food and food input (red cluster) embrace research on sugar cane juice and NCS bioactive compounds research, uses as a food input for jams, bakery products, juices or spirit beverages, input on dietary supplements, enhancing its conception from “healthy or traditional sweetener” to a key food functional and nutraceutical input in a wide variety of meals, as an ingredient in beverages and desserts, and as part of dietary regimes. Fifty research articles were related to this trend (17,8%^1^) highlighting the works related to: Beverages (García et al. 2017); granulated cane sugar and rice bran mix (Weerawatanakorn et al. 2017); whey protein (Ruano Uscategui et al. 2018); nutritional bar (Iuliano et al. 2019); vinegar (Chen et al., 2020); sport drink (Chew et al. 2020); jams (Cervera-Chiner et al. 2021); health-based spices and herbs (Singh and Rao 2021); and, 3D food printing (Thangalakshmi and Arora 2021).

Research Line 2–NCS as a healthy compound in pharmaceutic and cosmetic products (red cluster) comprises specialized research on NCS application on medicine, pharmacology, and cosmetic industries due to its phenolic, flavonoids, minerals, phosphates, anthocyanins, oligosaccharides, and vitamins content. Seven research documents (2,5%) present this emerging focus of NCS uses mainly due to policosanol content (Weerawatanakorn et al. 2019; Gayathry et al. 2021); phenolic content (Wijayanti et al. 2021); health effects (Chinnadurai 2017; Yang et al. 2020), and cytoprotective functions (Jaffé 2012; Pandiar et al. 2017).

Research Line 3–NCS production process optimization and greening: this line embraces process, equipment, and technological enhancement of sugar cane transformation on NCS. A total of 148 research documents (52,67%) comprise the highly developed research line: from milling process (Oktarini et al. 2019), passing through clarification (De Souza Sartori et al. 2017; Meerod et al. 2019), evaporation (Chantasiriwan 2017a, 2017b; Grewal and Kumar, 2021), concentration (Marie et al. 2020) and drying units process (Nishad et al. 2017, 2019). This trend promotes energy efficiency (Espitia et al. 2020; Gutiérrez-Mosquera et al. 2018; Srinvas et al. 2019; Venkata Sai and Reddy 2020), nutritional and sensorial (Kumar et al. 2017; Vera-Gutiérrez et al. 2019), and value added.

Research Line 4–NCS by-products promote diversification uses for sugar cane juice and NCS. A total of 47 research documents (15,30%) embrace by-products uses such as molasses (residue from clarification) and bagasse (residue from milling) into: nutritional blocks for animal feeding (Mendieta et al. 2020), alcohol-based chemicals (Raza et al. 2021), organic fertilizers (Gonçalves et al. 2021), nanocomposites (Bhattacharjee et al. 2020), bioethanol (Oliden et al. 2017), biohydrogen (Jayabalan et al. 2019), and spirit drinks (Aguiar et al. 2021).

#### Recent Advances on NCS Research

The previous framework contributes to the scientific landscape analysis of recent NCS research. Figure 4 (Suppletory Material) presents research advances in the last two years on each line proposed by Florez-Martínez et al. (2021). The cooccurrence network approach merges 298 topics into eight thematic clusters. Each cluster has been related to one or more lines.

##### Red Cluster–Antioxidants and Bioactive Compounds in NCS

This cluster is related to the uses of NCS as a functional and nutraceutical food and food input. Phenolic compounds and total phenolic content preservation from sugar cane juice contribute to enhanced antioxidant and antibacterial properties, with tricin and apigenin being the most abundant in granulated jaggery, muscovado sugar, light and regular jaggery blocks, cane honey, and brown sugar (Barrera et al. 2020). Other compounds, such as hydroxycinnamic acids (chlorogenic, caffeic, coumaric, and ferulic) and flavones (apigenin, tricin, and luteolin), in NCS have been identified as additives for green tea beverages, spicy beverages, and others (Anand et al. 2020; Dwiloka et al. 2020). The preservation of antioxidant bioactive compounds benefits from thermal processing techniques (Wang et al. 2020) and technologies instead of clarification process optimization (Zhu et al. 2020) and transformation processes such as spray drying (Alarcón et al. 2021), and neuroprotective properties of NCS in Parkinson’s disease patients have also been found (Cifuentes et al. 2021).

##### Blue Cluster–Uses of Sugar Cane Juice and Molasses

This cluster is related to both the uses of NCS as a functional and nutraceutical food and food input and NCS production by-products and coproducts. Molasses is one of the main by-products of NCS and other sugar cane juice derivatives used for ethanol production (Cruz et al. 2021) and is a main input for conceptual sugar cane biorefineries based on biocatalytic processes (Raut and Bhagat 2021). Furthermore, molasses could influence acrylamide and 5-hydroxylmethylfurfural formation and the sensory characteristics on NCS primarily due to Maillard reactions (Sung et al. 2020). Furthermore, sugar cane juice itself has been used as a nutraceutical additive for fruit and vegetable juices, jams, and syrups (Cervera-Chiner et al. 2021; Kumar et al. 2021a, b).

##### Green Cluster–Production Process Issues

This cluster includes topics related to NCS production process optimization and greening research lines. It includes the design, selection, and adoption of appropriate technologies for production lines comprising extraction, clarification, and by-product use for small producers (Beeram et al. 2020; Mayer et al. 2020; Nicolas Galvis et al. 2020). These technologies mainly focus on energy conservation and thermal efficiency (Velásquez et al. 2021; Venkata Sai and Reddy 2020), environmental performance and sustainability of production units (Quezada-Moreno et al. 2021), supply chain and commercial characteristics (Chen et al. 2021), and mathematical and simulation approaches (Baqueta et al. 2022).

##### Yellow Cluster–by-Product Valorization

This cluster is related to the NCS production by-product research line. NCS production processes generate two major by-products: bagasse biomass (milling activities) and molasses (clarification activities). Bagasse has been widely used as an alternative energy source, and current research has focused on sustainable use for both energy generation (Brunerová et al. 2020) and new uses in construction (Loganayagan et al. 2021). Furthermore, new technologies for energy efficiency and cogeneration (steam dryer) (Chantasiriwan 2021) and emissions reduction complete this cluster (Tyagi et al. 2021).

##### Purple Cluster–Flocculation Technologies

This cluster is related to NCS production process optimization and greening research lines and uses of NCS as a functional and nutraceutical food and food input. The clarification process of sugar cane juice is an important part of NCS production. This process is used for separating sediment and sugar cane juice by adding flocculant. Current research focuses on optimizing this process for to eliminate residues (Chantaruk et al. 2021; Teixeira et al. 2021) and preserve bioactive compounds, such as amino acids, flavonoids, polyphenols, esters, and acids (Zhu et al. 2020; Ge et al. 2021).

##### Cyan Cluster–Last Mile Clarification Technologies

This cluster is related to NCS production process optimization and greening research lines and the uses of NCS as a functional and nutraceutical food and food input. New clarification technologies contribute to both residue elimination and bioactive compound preservation instead of cost benefits and added value. Key technologies identified are ultrafiltration membranes (Vu et al. 2020), dielectric barriers (Manzoor et al. 2020), thermosonication (Adulvitayakorn et al. 2020; de Medeiros et al. 2021), and ceramic membranes (Zhu et al. 2020).

##### Orange Cluster–Key Pollutants

These research trends focus on two specific pollutants: acrylamides and furfurals. This research focuses on acrylamide formation due to low-humidity and high-temperature processes (Barón Cortés et al. 2021; Henao et al. 2021; Phaeon et al. 2021) and the presence and concentrations of colour enhancers, caramel, and molasses in sugar cane juice (Kobayashi et al. 2020; Sung et al. 2020). 5-Hydroxylmethylfurfural is another carcinomic compound of interest (Lee et al. 2020).

##### Brown Cluster–Commercial Products

NCS consumption and commercialization are enhanced by its antioxidant properties, nutritional properties, and bioactive molecules. These characteristics are enhanced by new clarification technologies (ceramic membranes and ultra-clarification) guaranteeing nutraceutical properties for beverages (Meng et al. 2021).

The relative importance of current scientific advances on the four NCS research lines was analysed using a thematic map. The map classifies research topics into four quadrants based on centrality (importance of the topic in the theme) and impact (density of the topic development). Figure 5 (Suppletory Material) presents a thematic map based on keyword occurrence.

- *Motor themes*: The upper right quadrant comprises the most relevant topics on both the development and importance of recent advances for NCS research. The sugar cane juice flocculation process; biomass use; and the preservation of antioxidants, flavonoids, polyphenols, and bioactive molecules encompass the research frontier.
- *Basic themes*: The lower-right quadrant represents the baseline topics, including unit operations in the NCS production process, such as clarification, evaporation, and fermentation (for bioproducts such as bagasse and molasses). Economic analysis focuses on sustainable development, reduced impacts, and quality factors (reduced acrylamide content).
- *Emerging or declining themes*: The lower-left quadrant consists of topics including value-added products such as functional foods, feed matrices, smart beverages, functional additives, and cosmetic and pharmaceutical ingredients. These topics encompass the diversification of the uses of NCS and future research pathways.
- *Niche themes*: The upper-left quadrant includes specialized topics such as biodegradation uses, phytochemical extraction, ethanol production, and biorefinery structure.

Finally, four articles represent novel uses of NCS: (i) Jaggery addition for bio-slurry production used for face mask (COVID-19) degradation (Patil et al. 2021), (ii) Extraction of the secondary metabolites of sugar cane juice used for alpha-amylase inhibitors and anti-COVID-19 effects (Wijayanti et al. 2021), (iii) NCS as an additive to exfoliant products (Bahari et al. 2021), and (iv) Use of NCS production wastes for fluorescent carbon nanomaterial “carbon dots” (El-Shafey 2021).

### Patent Analysis–Technological Development Phase 3, Results

The search strategy recovered 608 patents from 2017 to 2021, that were classified into the four research lines. Table 3 (Suppletory Material) presents the key patents identified using corpus data for each research line. A percentage of the patents are exclusively related to NCS, other are related to sugar cane juice, brown sugar, muscovado sugar, otherwise, all patents are related to cane sugar. A total of 389 patents were related to Research Line 1 (63,4%); 28 to Research Line 2 (5,6%); 96 to Research Line 3 (15,8%); and 95 to Research Line 4 (15,6%).

Related NCS patents comprise different contributions to the production process, foodstuff enrichment, feed stock design, and pharmaceutical and cosmetic uses. Furthermore, the modifier attributes in patents include the following: (i) Increasing the use of brown sugar in food, beverages, feeds, and other nutraceutical products; enhancing immunity against diseases; preserving bioactive compounds such as policosanols and flavonoids; and product quality characteristics including flavour, shape, palatability, and aroma; (ii) Decreasing production costs, the impurities of sugar cane juice, disease effects, fatigue, and moisture content; and (iii) Changing or stabilizing process variables such as temperature, pH, flavour, and taste (Suppletory Material, Fig. 6).

Value equation analysis presents the aggregate contribution of patents to problem solving. Efficiency factors influence the preservation of the natural flavours of sugar cane juice; the preservation of policosanols, minerals, flavonoids, vitamins, and other biocompounds; the energy use in each process stage; and the use of NCS as an additive to different product formulations for beverages, biscuits, sauces, jams, wines, and feedstuff. Harm comprises the added value of reducing and preventing diseases, environmentally friendly transformation processes, being free of pollutants, reducing moisture absorption, and being additive free and acid free. The interface relates the attributes of processes, products, and devices, such as easy absorption, digestion, control, processing, operations, storage, drinking, obtaining, and promotion. Finally, the cost component was related to NCS production efficiency (Suppletory Material, Fig. 6).

These analyses were complemented using title and abstract text and IPC codes on a cooccurrence network on VOSviewer. Figure 7 (see Suppletory Material) represents 15 thematic patent clusters that embrace the NCS technological development landscape (title and abstracts) and eight thematic clusters that embrace the normalized research area landscape (IPC codes).

- *Red Cluster–Sugar cane juice/violet cluster C05G3/00*: Sugar cane juice is the principal product of the milling process for NCS production and can be considered as an input for beverages, spirits, cakes, biscuits, cosmetics, biopharmaceuticals, and cosmetics. The patents in this cluster include those on the preservation of policosanols, flavonoids, and other bioactive compounds; filtration technologies including reverse osmosis membranes, microfiltration, and ultrafiltration membranes; and flocculants for absorption technologies. The IPC classification of these topics is related to the purification of sugar juices.
- *Green Cluster–Feed matrices/yellow cluster A23K10/00*: Using sugar cane juice milling (bagasse) and clarification (molasses) by-products and NCS for feed stuff addresses the concept of integral farm units. These by-products improve the organoleptic and nutritional quality of feed stuff, including enhancing the vitamin content, protein content, and palatability. The IPC family comprises formulation and production processes, including biochemical routes.
- *Blue, fuchsia, and orange clusters–healthy ingredients/red cluster A23L33*: NCS use as a food additive presents an advanced research field on products and process technologies, including the use of NCS in cereal formulations like flour and powder to increase mineral, vitamins, proteins, and bioactive compounds and as a natural sweetener for baked food, dairy-based beverages, fruit and tea juices, pulps, and nectars. The IPC family includes all inventions on modifying the nutritional quality of foods and dietetic products, their preparation, and their treatment.
- *Yellow Cluster–unitary operations/red cluster A23L2*: blue cluster C13B20/16: This cluster comprises the heating and clarification processes of NCS production focused on heat exchange, evaporators, energy efficiency, energy loss, exergy–emergy analysis, and multistage ceramic membranes. The IPC family comprises patents related to food preservation and physical separation methods.
- *Purple Cluster–bioactive compound extraction and use/green cluster A61k31*: This cluster is related to the clarification technologies for bioactive compound preservation, especially polyphenols. The IPC family comprises patents on preparations containing active ingredients.

The technological development of NCS includes all research lines identified in previous papers and promotes further projects based on recent advances.

### Colombian Market Trends and Niches–Phase 5, Results

The analysis of the growth of the main niche markets for Colombian NCS exports includes data for the last five years from the Legiscomex database. Principal target markets were identified, as well, the importer companies that cover 80% of the total market were classified into four types of niches: ethnic markets, functional foods, supplies for the food industry, and animal feeding. For each niche, we estimated the growth rate in the last five years and the total value of imports from Colombia.

The main importers of unrefined sugar (panela) from Colombia in the 2016–2021 period were the USA and Spain, with 17,433 tons and 13,967 tons, respectively, encompassing 68.9% of the total exports in the period. Other destinations were European countries such as France, Italy, and the UK. In Asia, South Korea was the principal importer; and in South America, Argentina and Chile were the principal importers (Suppletory Material, Fig. 8). In the USA and Spain, NCS focus on ethnic markets, and consumers come from Central and South America and India. The other European countries are markets with gourmet trends.

The largest niche market is the ethnic market, with 41.6 million dollars and 27,322 tons between 2016 and 2021. That market reported a growth rate over those 5 years of 43%. The second market of interest is inputs for functional products, which has a growth of 2,181% over 5 years and a value of 6.4 million dollars. This market is in Italy. The third niche is raw materials with a value of US$3.2 million and a growth rate of 7%. Finally, the fourth niche is animal feed, which has a growth rate of 39% but is still a small niche with a value of 0.5 million dollars. Additionally, Table 4 (Suppletory Material) shows the relations between the market niches and research lines for Colombian NCS exports.

### Holistic Analysis Technological Roadmap–Phase 5, Results

The NCS technological roadmap integrates the baseline results from previous phases on four specific layers (Fig. 9). Capacities layer comprises the national capacities on human resources, research projects, and institutions aligned with NCS research lines. Scientific research advances layer comprises the results and advances on each NCS research line from 2020 to 2021 (knowledge frontier). Technological products layer presents the patent mapping results that represent products related to each NCS research line. Market niches layer comprises the actual and potential market niches for Colombian NCS production based on foreign commerce data and world market trends. The relation between the elements in each layer and between layers defines the technology push or market pull routes for short-, middle-, and long-range terms (Suppletory Material, Fig. 9).

## Discussion

People worldwide are concerned about the rapid growth of new and noncommunicable diseases. Due to these facts, consumer demand for health-promoting foods has dramatically increased and the identification of new molecules that promote health and exhibit potential for technological applications (Paulo Farias et al., 2019). Furthermore, technological advancement reflects the demands for functional foods (Cai 2019; Kumar et al. 2021a, b). In general, the global demand for NCS market has increased because of the advancement of processing and production technologies, and people are aware of the importance of a healthy diet (Jaffé 2015). Although NCS is rich in sucrose, it is also a potential bioactive product with antioxidant activity. Colombian NCS market data indicate significant increase in the use of NCS as ingredient in food formulations.

One key challenge for NCS use is related to its production process, mainly due to final quality and safety (Aguilar-Rivera and Olvera-Vargas 2021). Therefore, the design, adoption, and adaptation of processing technologies include sugar cane juice extraction (milling and preservation), clarification (filtration technologies and natural clarifier substances), concentration evaporation (heat exchangers, thermoeconomics analysis, heat transfer modelling, exergy analysis, and crystallization), and packing (evapotranspiration and humidity control) (Aguilar-Rivera and Olvera-Vargas 2021; Meerod et al. 2019).

These technologies contribute to aligning products with specialized market niches, characterized by quality standards (Gómez and Espinosa 2017), the traceability of bioactive compounds (Meerod et al. 2020), being organic, engaging in fair trade, being ecological, providing origin appellation, and engaging in best manufacturing practices (García et al. 2017). Likewise, specialized market niches promote innovation in the food sector (juices, biscuits, sauces, desserts, preserves, herbal teas, wines, and jams) (Cervera-Chiner et al. 2021), feed sector (molasses, feed blocks, dietary supplements, and soil amendments) (Raza et al. 2021), energy sector (biomass and second-generation biofuels) (Raut and Bhagat, 2021), cosmetics (beauty creams, lipstick, scrub creams, and powder make up) (Chinnadurai 2017), and pharmaceuticals (Chinese traditional medicine, policosanol-based products, and antioxidant and anticancerogenic products) (Weerawatanakorn et al. 2019).

Furthermore, food processing technologies used in product patent design, such as spray drying, ultra-high-performance chromatography, cogeneration systems (Gamero et al. 2021), ceramic clarifiers (Shi et al. 2019), organic-base flocculants (Teixeira et al. 2021), and ultrafiltration membranes (Hakimzadeh et al. 2017), enhance NCS characteristics, such as flavour, shape, palatability, and aroma (Vera-Gutiérrez et al. 2019). Thus, patents provide valuable information for organoleptic potential conservation and bioactive compound preservation from sugar cane juice extraction until NCS conformation (liquid, block, powder, and granulated).

The technological roadmap proposed on this research aligns the four main research trends on NCS based on current functional foods baselines. These trends support the recent scientific advances as a main input for future research agendas, programmes, and projects formulation. Furthermore, trends enhance the current human, infrastructure, and technological capacities for R + D + I activities, reconfiguring both scientific and commercial knowledge on NCS value chain. Focusing capacities on each trend-advance topics, allows the design of technological products that converge on specialized market niches.

## Conclusions 

Colombian NCS has enormous potential in the identified market niches due to its quality and safety characteristics and the availability of production rates as an input and a direct use product. Bioactive compounds play a key role in technology design and adaptation through NCS production, and its preservation guarantees added value. Moreover, volatile compounds that confer NCS aroma, have the potential of use to design food and drinks with due to the sensorial fingerprint of NCS.

The roadmap itinerary presents the opportunity to construct research programmes on NCS that converge on innovative technologies development, and new market niches participation. Research programme 1, focused on quality enhancement (sensorial, design, and nutritional), of natural sweeteners and functional foods additives, comprises the preservation of biocompounds that enhance colour, aroma, flavour, and antioxidant properties, by using technologies of thermal efficiency, ultrafiltration, and drying. Research programme 2, focused on policosanol preservation comprises technologies across the production system that minimize losses on milling, clarification, evaporation, concentration, drying, and packing. Technologies like ceramic membranes, nano-membranes, and flocculation equipment, enhance both preservation and extraction of these biocompounds key input for ethnopharmacological products. Research programme 3, focused on NCS-Biorefineries that contributes to molasses (residues from clarification) and bagasse (residue from milling), exploitation for bioenergy, bioethanol, feed block matrices, diversifying traditional production units. Research programme 4, on secondary metabolites extraction and use, from sugar cane juice and clarification residues. Use of flocculation and evaporation technologies for acrylamide and furfurals formation or content reduction.

As a direct consumption product, the NCS traditional market niche supports the distribution and expansion of the product in different countries, supporting new uses and consumption trends. NCS uses as an input for to replace the sweetening of refined sugar, maltitol, xylitol, sucralose, and even stevia due to its minerals, flavonoids, polyphenols, vitamins, and trace elements and to establish organic, kosher, and healthy brands. Furthermore, NCS uses, as a bioactive compound in health and cosmetic products by conferring symbiotic properties with other natural ingredients. As a by-product, molasses and bagasse promote the concept of a circular economy in NCS production units.

The design of future research agendas on functional foods; bioactive compounds, primarily and secondary metabolites; added value products and health-cosmetic ingredients need to focus on short-, middle-, and long-term scenarios based on technology push and market pull analysis, balanced research capacities, diversified financial budgets and interactions between academia, research centres and food, feed, pharmaceutical, and cosmetic industries.

Finally, the methodological design proposed on this research could be implemented on all NCS producer countries, for research agenda definition and specific technological roadmaps design. Furthermore, the methodology can be adapted for other functional foods analysis.

## Supplementary Information

Below is the link to the electronic supplementary material.

Supplementary file1 (DOCX 6536 kb)

## Footnotes

1 Thirty-two documents were excluded due its low alignment with NCS research (11,38%).
