# Supplementary material for: Key Drivers for Non-Centrifugal Sugar Cane Research, Technological Development, and Market Linkage: A Technological Roadmap Approach for Colombia
Source: Sugar Tech. 2022 Sep 1;25(2):373–85. doi: 10.1007/s12355-022-01200-9 (PMC9434537; doi:10.1007/s12355-022-01200-9)
Supplement: Supplementary file 1 — Supplementary file1 (DOCX 6536 kb) [file 12355_2022_1200_MOESM1_ESM.docx]

**Suppletory material**


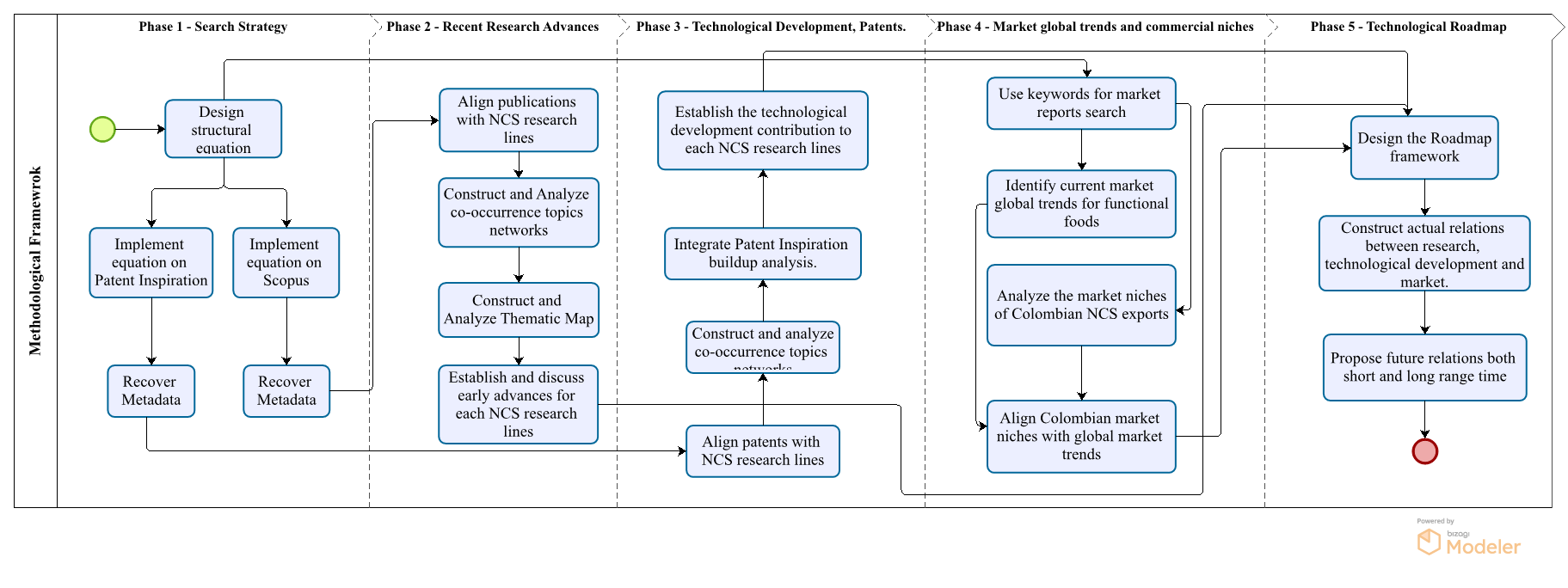
Fig. 1 Methodological framework

Source. Authors on Bizagi®

Table 1. Search strategy construction

| Database | Scope | Keywords | Restrictions |
| --- | --- | --- | --- |
| Scopus® | Scientific advances on NCS research | Group1: technical, common, and relative terms for NCS.  Group 2: technical terms related to food science concepts, methods, technologies used on NCS production. | Timelapse: 2017-2021  Exclude Brown Sugar |
| Scopus® | Scientific advances on functional foods | Group1: technical, common, and relative terms for functional foods. | Timelapse: 2017-2021 |
| PatentInspiration® | Technological development advances on NCS uses | Group1: technical, common, and relative terms for NCS.  Group 2: technical terms related to food science concepts, methods, technologies used on NCS production. | Timelapse: 2017-2021  Include brown sugar |
| Legiscomex® | Trade data for Colombian NCS | NCS National Specific tariff heading 1701130000: Product: 1701130000 Cane sugar, in solid state, without the addition of flavouring or colouring, obtained without centrifugation, with a sucrose content 69 ° to 93 °, containing only natural anhedral microcrystals | Timelapse: 2017-2021 |
| EMIS® | Global market reports for functional foods | Functional foods  Nutraceutic foods  Healthy foods | Timelapse: 2017-2021 |

Source: Authors®

Table 2. Search equations results

| N° | Search equation | Database | Registries |
| --- | --- | --- | --- |
| 1 | TITLE-ABS-KEY ( ( "non-centrifuged sugar" OR "non-centrifugal sugar" OR "non-centrifugal cane sugar" OR " non-centrifugal sugarcane" OR "non-centrifuged cane sugar" OR "panela" OR "jaggery" OR "unrefined whole cane sugar" OR "muscovado sugar" OR "raw sugar" OR "sugarcane juice" OR "evaporated sugarcane juice" OR "raw sugar cane" OR “brown sugar”^[[1]](#footnote-1)^ ) AND ( "acid treatment" OR "sodium hydrosulphite treatment" OR "concentration process" OR "evaporation process" OR "milling process" OR "clarification process" OR "filtration process" OR "spray process" OR "spray dry*" OR "sucrose content" OR "glucose content" OR "fructose content" OR "phenol content" OR "flavonoid* content" OR "mineral* content" OR "vitamin* content" OR "nutritional compound*" OR "nutritional propert*" OR "nutraceutical propert*" OR "antioxidant propert*" OR "functional properties" OR "health* properties" OR "health* food" OR "animal feed" OR "nutraceutical product" OR "natural sweeteners" OR "traditional food" OR "food additives" OR "beverage additives" OR "cosmetic additives" OR "pharmaceutic* additives" OR "innova* products" OR "production process*" OR "technolog*" OR "value added" OR "powder product*" OR "environmental indices" OR "aroma compound*" OR "smell compound" OR "acrylamide" OR "heat exchanger*" OR "computational fluid dynamics" OR "physicochemical propert*" OR "physical propert*" OR "thermophysical propert*" OR "energy efficiency" OR "exergy" OR "thermal performance" OR "policosanol*" ) ) AND PUBYEAR > 2017 | Scopus® | 281 |
|  |  | PatentInspiration® | 608 |
| 2 | TITLE-ABS-KEY (("functional foods" OR "functional beverages" OR "functional ingredients" OR "functional additives" OR "functional feeds") AND ("trends")) AND PUBYEAR > 2017 | Scopus® | 573 |
| 3 | TITLE-ABS-KEY (( "functional foods" OR "functional beverages" OR "functional ingredients" OR "functional additives" OR "functional feeds" ) AND ( "Non-centrifuged sugar" OR "non-centrifugal sugar" OR "non-centrifugal cane sugar" OR " non-centrifugal sugarcane" OR "non-centrifuged cane sugar" OR "panela" OR "jaggery" OR " unrefined whole cane sugar" OR "muscovado sugar" OR "raw sugar" OR "sugarcane juice" OR "evaporated sugarcane juice" OR "raw sugar cane" ) ) | Scopus® | 19 |
| 4 | ("functional foods" OR "functional beverages" OR "functional ingredients"  OR "functional additives" OR "functional feeds") | Emis® | 12 |
| 5 | 701130000 - CANE SUGAR MENTIONED IN SUBHEADING NOTE 2 TO THIS CHAPTER | Legiscomex® | 4,285 |

Source. Authors


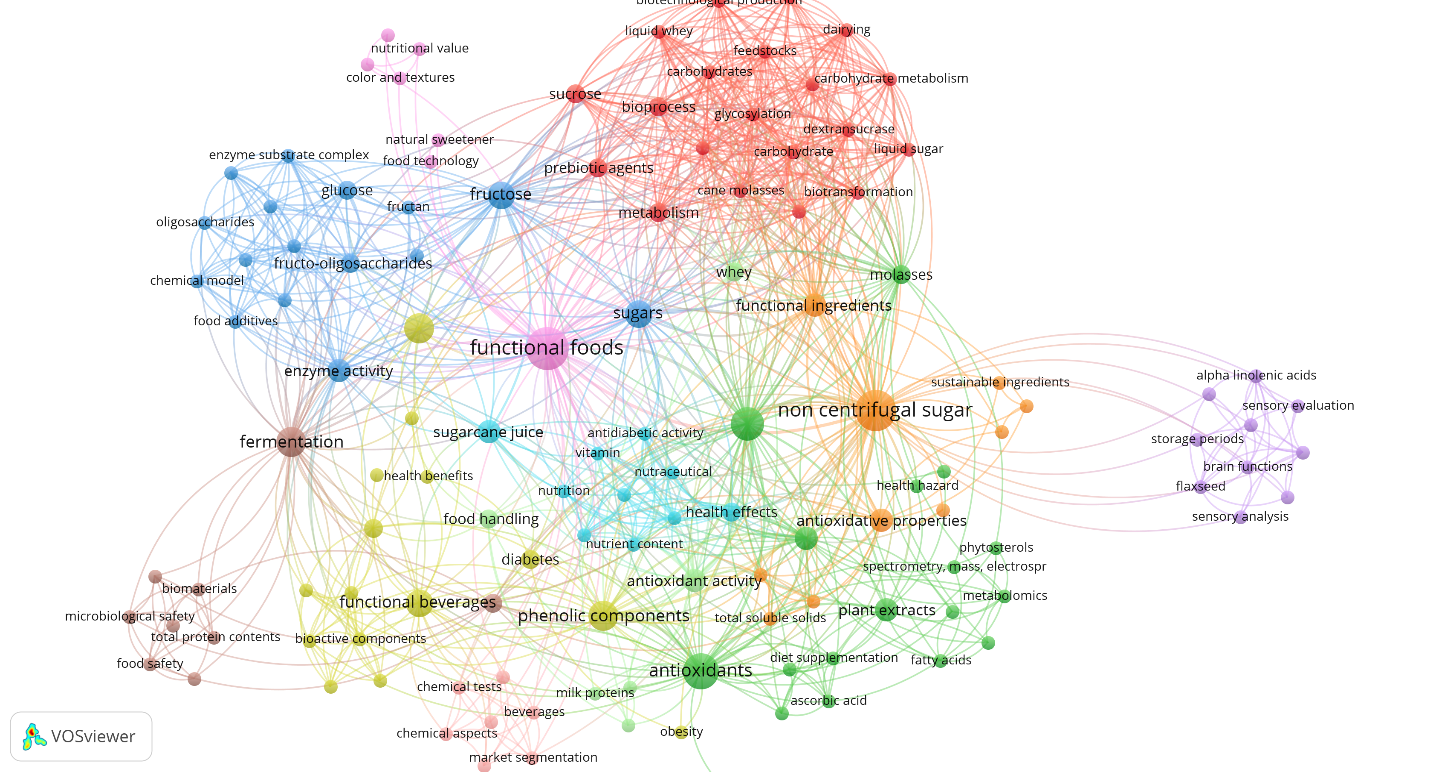

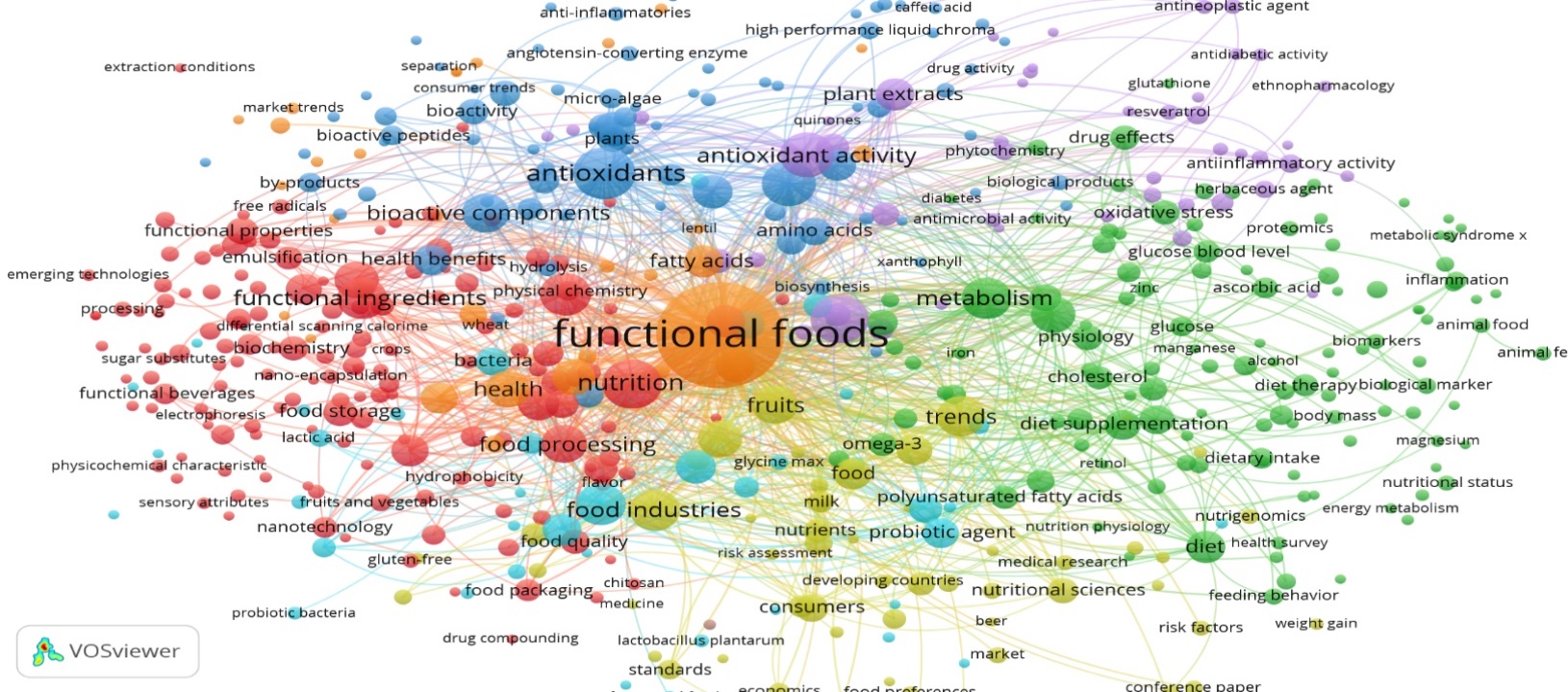
Fig. 2 Functional food scientific landscape 2017-2021 and NCS research as a functional food

Source: Authors based on Scopus data. December 2021. Processing software VOSviewer 1.6.17


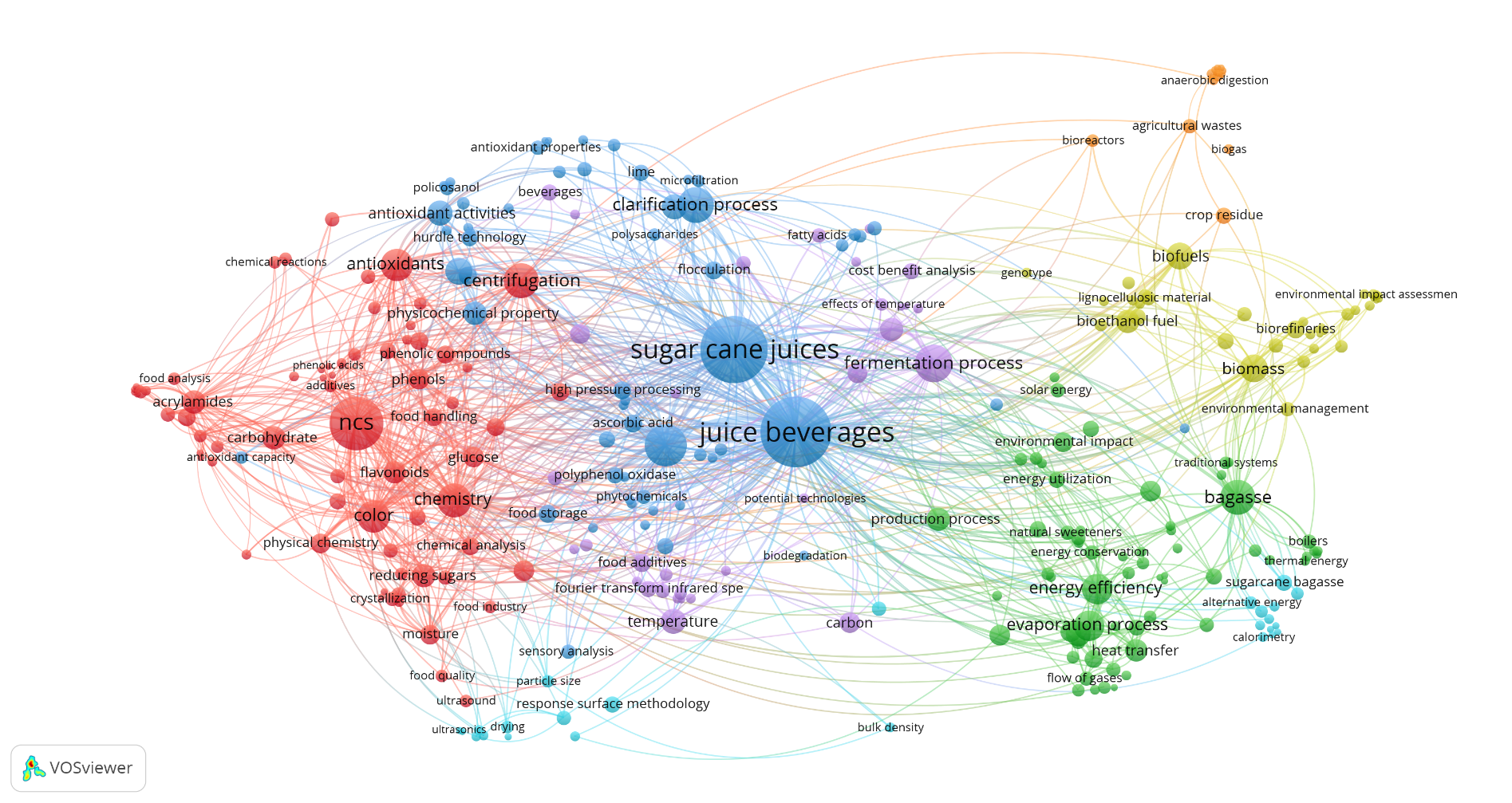
Fig. 3 NCS recent scientific trends landscape 2017-2021

Source. Authors based on Scopus data. December 2021. Processing software: VOSviewer 1.6.17


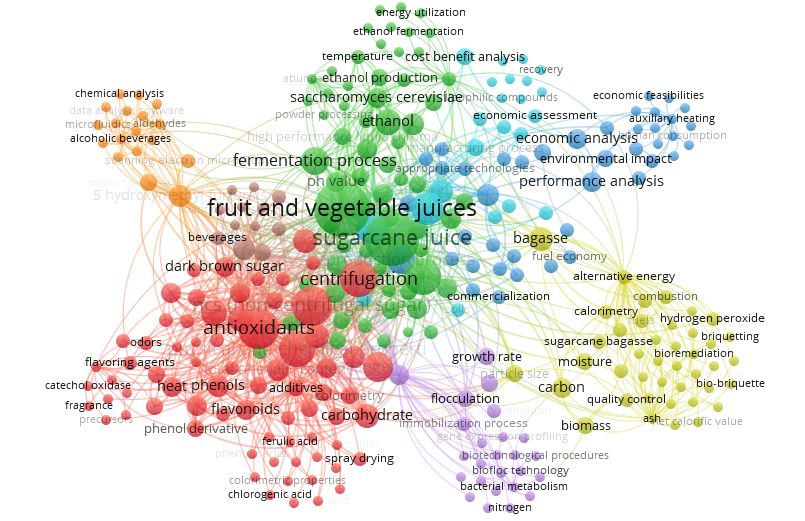
Fig. 4 NCS recent scientific advances landscape 2020-2021

Source. Authors based on Scopus data. December 2021. Processing software: VOSviewer 1.6.17.

Fig. 5 NCS thematic coupling map


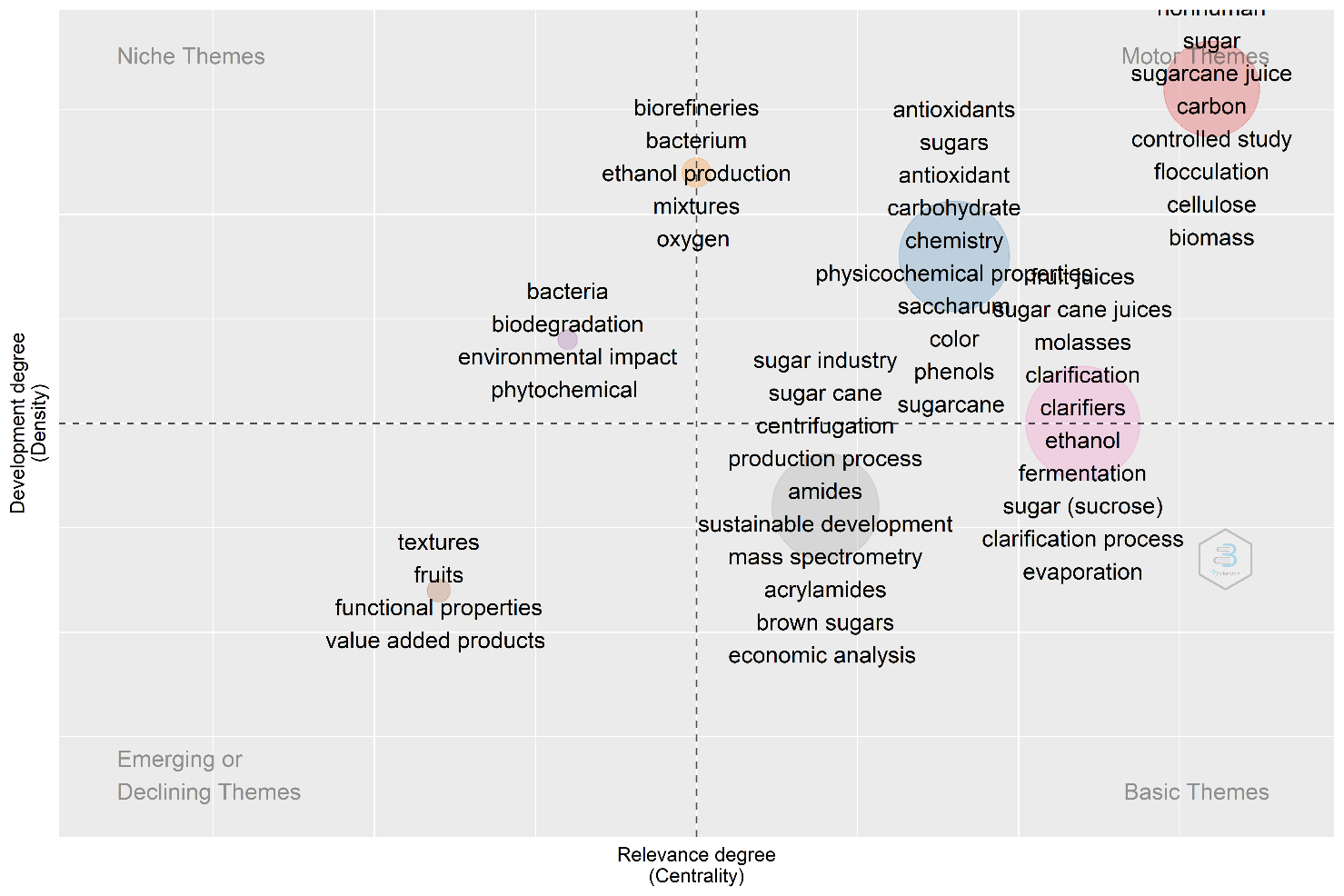


Source. Authors based on Scopus data. December 2021. Processing software: Bibliometrix 3.1.4.

Table 3. Patent classification on research lines

| **Research Line** | **Key patents** | **IPC and key topics** | **Patent number** | **Scope** |
| --- | --- | --- | --- | --- |
| NCS as a healthy compound in pharmaceutic and cosmetic products (32 patents) | - Polyphenol-rich brown sugar moisturizing mask and preparation method thereof (2021) | - A61K8/9794, A61K8/34, A61K8/60, A61Q19/00, and A61Q19/02 - Sugarcane varieties rich in polyphenol - Membrane filtration technologies | [CN112545956A](https://worldwide.espacenet.com/patent/search?q=CN112545956A) | Brown sugar and NCS |
|  | - Antioxidant and anticarcinogenic sugarcane extract and production thereof (2020) | - Unsaturated aldehydes having chain lengths between 44 and 78 carbon atoms - Beneficial sugarcane products and by-products - Supercritical fluid extraction | [WO2020256570A2](https://worldwide.espacenet.com/patent/search?q=WO2020256570A2) | NCS, Brown sugar and other cane juice products |
|  | - Polyphenol-rich brown sugar freckle-removing mask and preparation method thereof (2021) | - Brow sugar compounds - Polyphenol sugar cane varieties - A61K8/9789, A61K8/34, A61K8/73, A61K8/92, A61K8/9728, A61Q19/00, A61Q19/02, A61Q19/08 | [CN112472645A](https://worldwide.espacenet.com/patent/search?q=CN112472645A) | Brown sugar and NCS |
|  | - An application of a sugarcane cell water beverage containing sugar in treatment of diabetes mellitus (2021) | - Control sugar blood content - A61K36/899, A23L2/02, A23L2/04, A23L33/105, A61P3/10 | [CN109200213A](https://worldwide.espacenet.com/patent/search?q=CN109200213A) | Sugar cane juice |
| NCS production by-products | - Brown sugar and red date single-vitamin nutrition-fortified flowering steamed buns and production process thereof (2021) | - Flowering and nutritional mixture - A23L7/104, A23L19/00, A23L33/10, A23L33/125, A23L33/15 | [CN112890092A](https://worldwide.espacenet.com/patent/search?q=CN112890092A) | Brown Sugar and NCS |
|  | - Amino acid active ingredient containing total nutrient liquid fertilizer for plants and production process therefor (2020) | - C05G3/80 and C05F17/00 - Biotype total nutrient liquid fertilizer - Supplementing nutrients for plant growth - Raw brown sugar | [CN110803964A](https://worldwide.espacenet.com/patent/search?q=CN110803964A) | Brown Sugar and NCS |
|  | - System and method for producing ethanol from sugarcanes (2020) | - C12P7/10, C07C29/76, C07C31/08, C12M1/40 - Production process - Unit operations - Bioethanol | [CN110760548A](https://worldwide.espacenet.com/patent/search?q=CN110760548A) | Sugarcane juice |
|  | - Safety and environmentally friendly mosquito-repelling enzymes and preparation method thereof (2019) | - A01M29/12 - Sustainable products - Derived enzyme from raw products - Brown sugar | [CN110367237A](https://worldwide.espacenet.com/patent/search?q=CN110367237A) | Brown sugar and NCS |
|  | - Novel granular organic fertilizer production process and organic fertilizer prepared with same (2018) | - C05F11/08, C05F17/00, and C05G5/00 - Organic fertilizers - Inoculants - Mechanical granulation - Brown sugar | [CN107602168A](http://www.patentinspiration.com/redirect?url=/patent/CN107602168A) | Brown sugar and NCS |
| NCS production process optimization and greening (123) | - Method for quantitatively detecting acrylamide in brown sugar (2021) | - Acrylamide detection technique - Liquid chromatography ultraviolet - G01N30/88 | [CN112798725A](https://worldwide.espacenet.com/patent/search?q=CN112798725A) | Brown sugar and NCS |
|  | - A production method of liquid brown sugar with sugarcane as raw material (2021) | - C13B30/00, C13B10/02, C13B20/16, and C13B50/00 - Foaming - Clarification - Shape stability | [AU2021102297A4](https://worldwide.espacenet.com/patent/search?q=AU2021102297A4) | Brown sugar and NCS |
|  | - Method for processing raw sugarcane to maximise the preservation of policosanols during the production of a natural sugarcane juice-based product (2021) | - Flocculating - Policosanol - Sugarcane juice clarification - A61K31/045, A61K36/899, A61K2236/15, A61K2236/31, A23L33/105, and C13B10/003 | [ZA202002161B](https://worldwide.espacenet.com/patent/search?q=ZA202002161B) | Brown sugar and NCS |
|  | - Production process for brown sugar with high melanoidin content (2020) | - C13B50/00, C13B20/16, and C13B25/00 - Filter screen - Concentration - Melanoidins | [CN111424121A](https://worldwide.espacenet.com/patent/search?q=CN111424121A) | Brown sugar and NCS |
|  | - Systems and methods for preserving nutrients during the production of syrups and powders from sugarcane using cold technology and products containing the same (2020) | - Cold processing pipeline - Bioactive compounds preservation - C13B10/02, C13B25/00, C13B50/00 | [US2020270710A1](https://worldwide.espacenet.com/patent/search?q=US2020270710A1) | Brown sugar and NCS |
|  | - A process for preparation of flavour coated jaggery (2019) | - Coated Jaggery particles - Low moisture content - Low moisture absorbent capacity - A23L29/30, A23L27/20 | [WO2019211678A1](https://worldwide.espacenet.com/patent/search?q=WO2019211678A1) | NCS |
|  | - Brown sugar production process combining traditional brown sugar production process and modern brown sugar production process (2017) | - C13B5/00, A23G3/48, C13B10/00, C13B10/02, C13B10/06, C13B20/16, and C13B25/04 - Natural gas plum-blossom stove for heating - Granulating vessel - Nutrient conservation | [CN107267669A](https://worldwide.espacenet.com/patent/search?q=CN107267669A) | Brown sugar and NCS |
|  | - Production line of rich polyphenol brown sugar (2017) | - C13B20/16, C13B10/02, C13B20/02, C13B25/00, C13B30/00, C13B30/02, and C13B50/02 - Utility model - Production line - Ceramic membrane | [CN205974554U](https://worldwide.espacenet.com/patent/search?q=CN205974554U) | Brown sugar and NCS |
| NCS uses as a functional and nutraceutical food and food input | - Mixing and concentrating device for producing brown sugar ginger tea (2021) | - B01D1/00, A23F3/34, and B01D1/30 - Mineral content - Tea enhancement - Sweetener | [CN212548319U](https://worldwide.espacenet.com/patent/search?q=CN212548319U) | Brown sugar and NCS |
|  | - Preparation method of insect protein source biological feed (2021) | - Protein feed - Selenium - Brown sugar - A23K10/12, A23K10/26, A23K10/30, A23K20/142, A23K20/158, A23K20/163, A23K20/20 | [CN112753850A](https://worldwide.espacenet.com/patent/search?q=CN112753850A) | Brown sugar and NCS |
|  | - Granules made from pear syrup, brown sugar and ginger tea and the production process of granules (2020) | - A23F3/34 - Granulation - Taste enhancer - Physicochemical indices | [CN111802493A](https://worldwide.espacenet.com/patent/search?q=CN111802493A) | Brown sugar and NCS |
|  | - Nutrient and nourishing brown sugar product (2019) | - A23G3/48; andA23G3/36 - Mixing - Jelly - Nourishing function | [CN111227093A](https://worldwide.espacenet.com/patent/search?q=CN111227093A) | Brown sugar and NCS |
|  | - The composition of a natural alternative sweetener from stevia rebaudiana and muscovado sugar (2019) | - A23L27/00, A23L27/30, and A23L33/10 - Mixing - Powder - Taste enhancement | [PH22018000817U1](https://worldwide.espacenet.com/patent/search?q=PH22018000817U1) | Brown sugar and NCS |
|  | - Solid drink containing brown sugar, fructus lycii and algae (2018) | - Food production - High nutrient values - A23L2/39, A23L2/52, A23L2/60, A23L33/00 | [CN109007513A](https://worldwide.espacenet.com/patent/search?q=CN109007513A) | Brown sugar and NCS |
|  | - Preparation method of selenium-enriched ancient brown sugar (2017) | - A23G3/48, A23G3/36, and C13B50/00 - Enriched brown sugar - Selenium sweet potato - Cost reduction | [CN107156412A](https://worldwide.espacenet.com/patent/search?q=CN107156412A) | Brown sugar and NCS |

Source. Authors based on PatentInspiration® data retrieved in December 2021

Figure 6. NCS patent modifiers, and value equation.


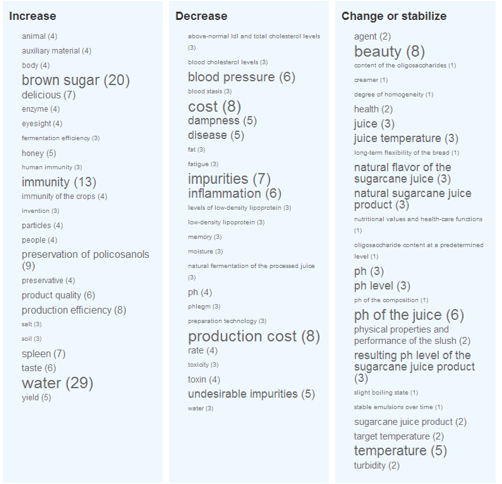

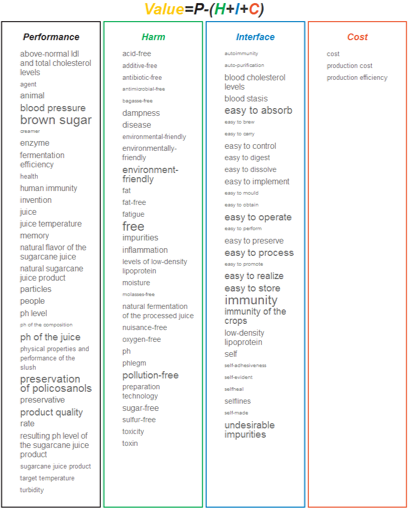


Source. Constructed on PatentInspiration® in December 2021


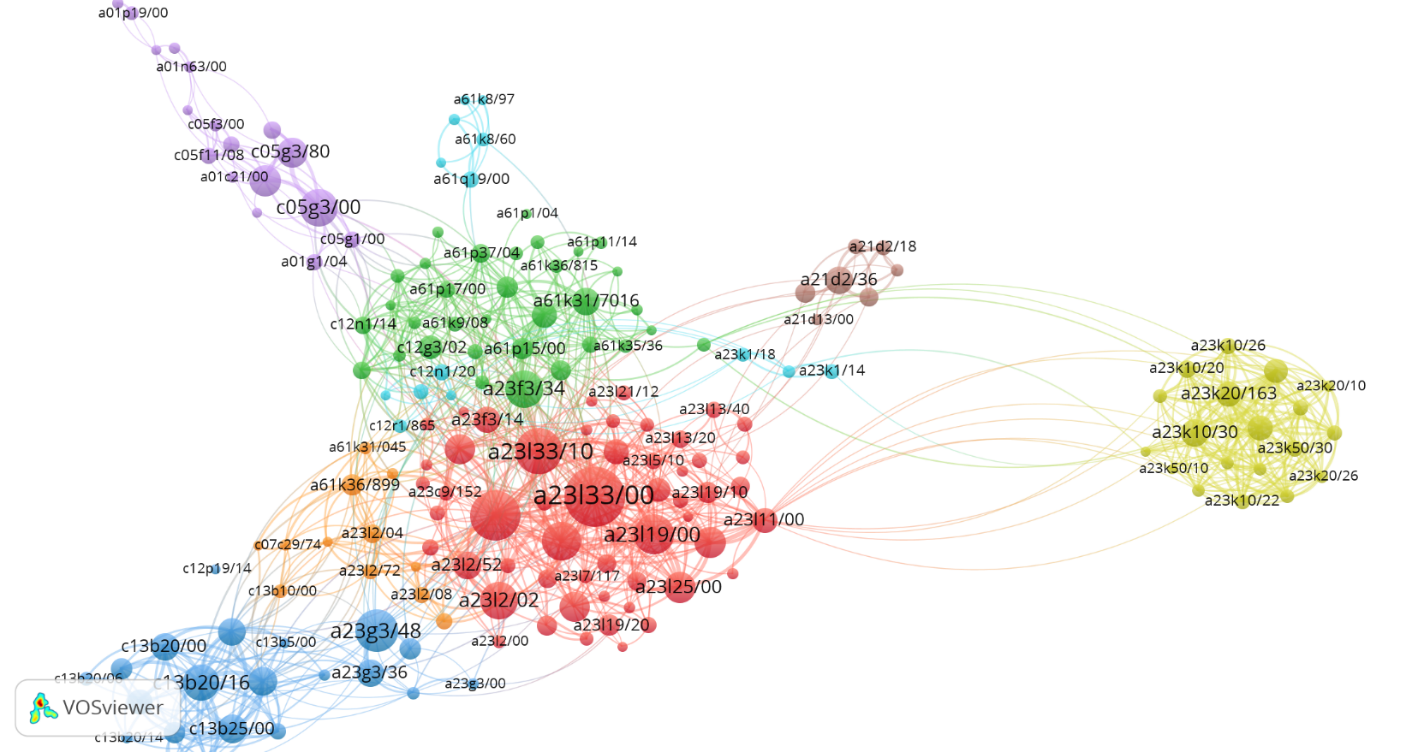

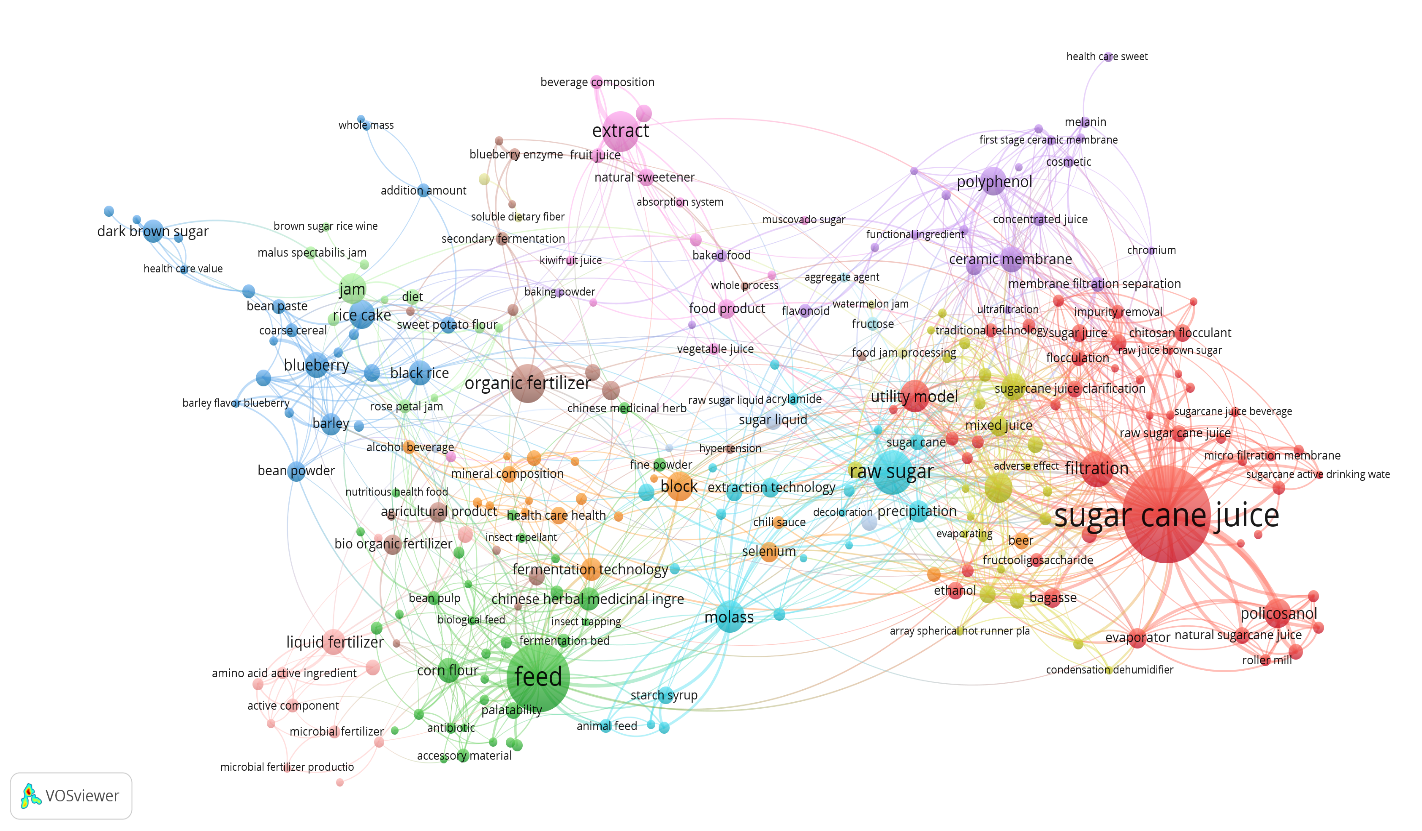
Fig. 7 NCS-related patent landscape

Source. Own elaboration using VOSviewer® and based on PatentInspiration® data consulted in December 2021.

Figure 8. Top 10 Colombian NCS importers, 2016-2021.

Source. Own elaboration based on Legiscomex® database. Retrieved September 2021. Processing software: Excel®.

Table 4. Colombian market niches, research lines, key partners, and destination countries

| **Market Niche** | **Research lines** | **Top Companies** | **Export destination** | **Value (USD)** | **Total Market 2015-2021 (USD)** |
| --- | --- | --- | --- | --- | --- |
| Supplies for food industry | NCS production by-products | B&M Sugar Products, Ltd. | France, Spain, the United Kingdom, and Czech Republic | 2,089,313 | 3,235,595 |
|  |  | Ensicro S.R.L | Argentina | 595,371 |  |
|  |  | Pronatec BV | Netherlands | 550,911 |  |
| Functional foods | Functional and nutraceutical food | Laboratoris Almond | Spain | 4,816,074 | 6,437,118 |
|  |  | Everton Spa | Italy | 1,621,045 |  |
| Ethnic Foods Market | NCS production by-products | Goya Foods | Spain and USA | 6,771,095 | 41,633,420 |
|  |  | Grace Kennedy Foods | USA and Canada | 3,048,319 |  |
|  |  | Imepex Sociedad Por Acciones Simplificada | Spain and Italy | 2,469,980 |  |
|  |  | Corporacion Panelera Dona Panela | Spain, Australia, Argentina, South Korea, France, Italy, and USA | 2,174,639 |  |
|  |  | Goex LLC | Spain and USA | 2,134,383 |  |
|  |  | Auno Co. Ltd. Mr. Kwon Jaewon | South Korea | 1,920,449 |  |
|  |  | Other | Other | 23,114,557 |  |
| Animal feeding | NCS production by-products | Bio Alimentación Colombia | Spain | 499,620 | 499,620 |

Source. Own elaboration based on Legiscomex® database. Retrieved September 2021. Processing software: Excel®.


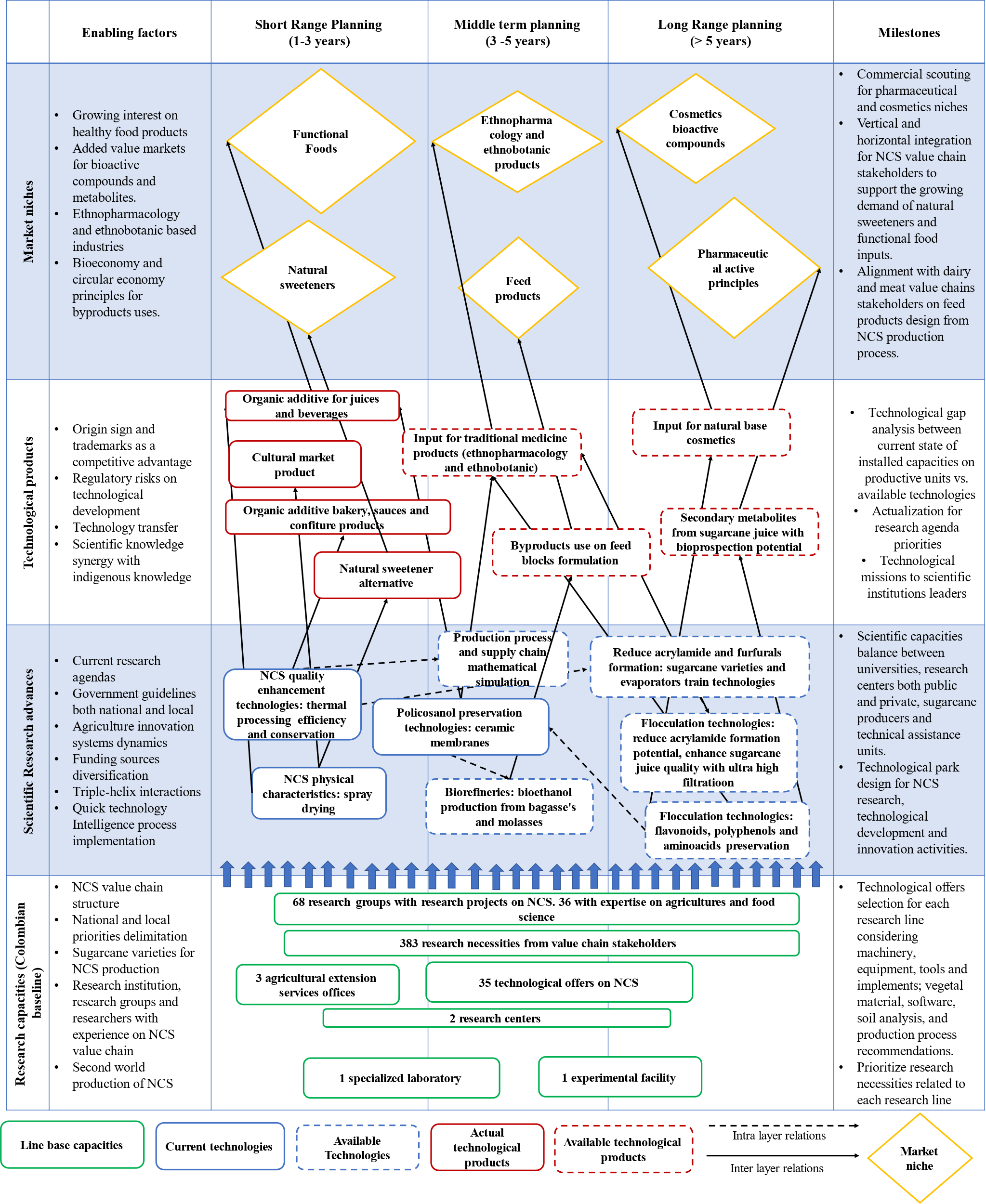
Fig. 9 NCS technological roadmap

Source. Own elaboration

1. Keyword “Brown sugar” was excluded on Scopus® search and included on PatentInspiration® database [↑](#footnote-ref-1)
